# Comprehensive assessment showed no associations of variants at the *SLC10A1* locus with susceptibility to persistent HBV infection among Southern Chinese

**DOI:** 10.1038/srep46490

**Published:** 2017-04-21

**Authors:** Ying Zhang, Yuanfeng Li, Miantao Wu, Pengbo Cao, Xiaomin Liu, Qian Ren, Yun Zhai, Bobo Xie, Yanling Hu, Zhibin Hu, Jinxin Bei, Jie Ping, Xinyi Liu, Yinghua Yu, Bingqian Guo, Hui Lu, Guanjun Liu, Haitao Zhang, Ying Cui, Zengnan Mo, Hongbing Shen, Yi-Xin Zeng, Fuchu He, Hongxing Zhang, Gangqiao Zhou

**Affiliations:** 1 School of Life Sciences, Tsinghua University, Beijing, China; 2 State Key Laboratory of Proteomics, Beijing Proteome Research Center, Beijing Institute of Radiation Medicine, Beijing, China; 3 National Engineering Research Center for Protein Drugs, Beijing, China; 4 National Center for Protein Sciences Beijing, Beijing, China; 5 State Key Laboratory of Oncology in South China, Collaborative Innovation Center for Cancer Medicine, Sun Yat-sen University Cancer Center, Guangzhou, China; 6 Department of Laboratory Medicine, Sun Yat-sen University Cancer Center, Guangzhou, China; 7 Center for Genomic and Personalized Medicine, Guangxi Medical University, Nanning, Guangxi, China; 8 Department of Epidemiology and Biostatistics, MOE Key Laboratory of Modern Toxicology, School of Public Health, Nanjing Medical University, Nanjing, China; 9 State Key Laboratory of Oncology in Southern China, Guangzhou, China; 10 Affiliated Cancer Hospital of Guangxi Medical University, Nanning, Guangxi, China

## Abstract

The sodium taurocholate cotransporting polypeptide (NTCP) encoded by *SLC10A1* was recently demonstrated to be a functional receptor for hepatitis B virus (HBV). The role of *SLC10A1* polymorphisms, particularly the Ser267Phe variant (rs2296651) in exon 4, has been frequently investigated in regard to risk of persistent HBV infection. However, these investigations have generated conflicting results. To examine whether common genetic variation at the *SLC10A1* locus is associated with risk of persistent HBV infection, haplotype-tagging and imputed single nucleotide polymorphisms (SNPs) were assessed in two case-control sample sets, totally including 2,550 cases (persistently HBV infected subjects, PIs) and 2,124 controls (spontaneously recovered subjects, SRs) of Southern Chinese ancestry. To test whether rare or subpolymorphic *SLC10A1* variants are associated with disease risk, the gene’s exons in 244 cases were sequenced. Overall, we found neither SNPs nor haplotypes of *SLC10A1* showed significant association in the two sample sets. Furthermore, no significant associations of rare variants or copy number variation covering *SLC10A1* were observed. Finally, expression quantitative trait locus analyses revealed that SNPs potentially affecting *SLC10A1* expression also showed no significant associations. We conclude that genetic variation at the *SLC10A1* locus is not likely a major risk factor of persistent HBV infection among Southern Chinese.

Hepatitis B virus (HBV) infection is a major infectious disease with more than 250 million chronic carriers worldwide, with potential to cause chronic hepatitis, liver cirrhosis, hepatocellular carcinoma (HCC) and death^1^. Persistent HBV infection or HBV clearance is influenced by complex factors of viral, environmental, and genetic components, including HBV genomic variability, host age and sex, concurrent infection with the hepatitis C virus, hepatitis D virus, and human immune deficiency virus^2^,^3^. The segregation analyses and twin studies have provided strong evidence that host genetic factors play a role in determining the persistence of HBV infection^4^,^5^. Recently, several genome-wide association studies (GWASs) by us and other researchers have identified single nucleotide polymorphisms (SNPs) at nine loci linking genetic susceptibility to persistent HBV infection in populations of Asia ancestry, including *HLA*-*DP* (index rs3077 and rs9277535), *HLA*-*DQ* (rs2856718 and rs7453920), *HLA*-*C* (rs3130542), *EHMT2* (rs652888), *TCF19* (rs1419881), *CFB* (rs12614) and three non-HLA loci *INTS10* (rs7000921), *UBE2L3* (rs4821116) and *CD40* (rs1883832)^6^,^7^,^8^,^9^,^10^. However, the susceptibility to infectious diseases such as HBV infection is considered to be determined at multiple functional levels^11^, suggesting additional genetic components need to be discovered.

Sodium taurocholate cotransporting polypeptide (NTCP), encoded by the gene solute carrier family 10 (sodium/bile acid cotransporter family), member 1 (*SLC10A1*) located on chromosome 14, is the founding member of the SLC10 family of solute carrier proteins. NTCP is a multiple transmembrane glycoprotein presumed to span the cellular membrane up to 10 times with small extracellular loops^12^. NTCP is mainly expressed in hepatocyte, where it acts as a hepatic Na^+^/bile acid symporter and is responsible for cotransportation of sodium and bile acids across cellular membranes to maintain the enterohepatic circulation of bile acids^13^. Recently, Yan *et al*. have reported that NTCP is a functional receptor for HBV and its satellite hepatitis D virus (HDV)^14^. They used a combination of advanced purification and mass spectrometry analysis to show that the NTCP on the surface of the primary liver cells from treeshrews interacts with the pre-S1 domain in HBV. When the *SLC10A1* gene was silenced in liver cells of both human and treeshrew origin, HBV infection was greatly reduced. Moreover, transfecting HepG2 cells with *SLC10A1* from humans and treeshrews would make these cells gain susceptibility to HBV and HDV infection. Similarly, although monkey and mouse are not susceptible to HBV, replacing amino acids 157–165 of the monkey NTCP or 84–87 of the mouse NTCP with their human counterparts was enough to make the NTCP a functional receptor for the viruses^14^,^15^,^16^.

As NTCP is the first and the only one confirmed to be functional in supporting viral infection among a number of HBV receptor candidates reported up to now, it is expected that the genetic variation within *SLC10A1* could influence the effects of gene products, which in turn results in genotype-dependent differences in susceptibility to persistent HBV infection. Considerable attention has been focused on a common polymorphism rs2296651 (p.Ser267Phe, c.800G > A) in exon 4 of the *SLC10A1* gene. The Ser267Phe variant has normal cell surface expression but results in loss of bile salt uptake and HBV receptor function^17^,^18^,^19^,^20^,^21^. However, previous association studies on Ser267Phe and HBV infection have generated conflicting results^21^,^22^,^23^,^24^. Furthermore, candidate regulatory variants, haplotype-tagging SNPs (htSNPs) or variants across the entire region of *SLC10A1* have been assessed to determine the effects of them on risk of HBV infection^24^,^25^,^26^. Overall, evidence for the association of these variants with HBV infection is mixed, and need to be investigated further in independent and large cohorts as replication is an important approach to verify any significant genetic association findings^27^,^28^. For example, as the minor allele frequency of Ser267Phe is ~7.5% in people of Chinese origin^21^,^22^,^23^,^24^, the association between the variant and risk of persistent HBV infection, if confirmed or negated, bears important public health implications for molecular targeted treatment and prevention of the disease.

In the present study, we performed a comprehensive examination of variants at the *SLC10A1* locus in two case-control populations, totally including 2,550 cases (persistently HBV infected subjects, PIs) and 2,124 controls (spontaneously recovered subjects, SRs) of Southern Chinese ancestry. Overall, we found no evidence that genetic variation at *SLC10A1* is likely to be a major risk factor of persistent HBV infection.

## Results

To comprehensively detect variants at the *SLC10A1* locus conferring susceptibility to persistent HBV infection, we used two case-control populations, namely the Sample Set 1 (Guangxi population) and the Sample Set 2 (GWAS population) (Supplementary Table 1A). The Sample Set 1 contains 1,299 PIs (cases) and 1,067 SRs (controls) recruited from Guangxi, China^10^. For the Sample Set 2, we re-used published GWAS data among totally 12,027 individuals of Chinese ancestry^29^,^30^,^31^,^32^, and finally obtained five independent GWAS sub-populations totally including 1,251 PIs and 1,057 SRs by screening for HBV markers as previously reported (Methods)^10^. There was no significant difference between cases and controls in terms of sex and mean age distribution in the two sample sets (all *P* values > 0.05, Supplementary Table 1A).

### Association between SNPs at the *SLC10A1* locus and persistent HBV infection

Using the genotype data from Han Chinese in Beijing (CHB) and Han Chinese South (CHS) samples of the 1000 Genomes Project (http://browser.1000genomes.org/index.html), we reconstructed one major haplotype block totally ~80-Kb in length covering the entire *SLC10A1* gene at chromosome 14q24.1 (chr14:70,206,417–70,287,166). This dataset derived from the 1000 Genomes Project contains nearly 7.5 times more common SNPs and low-frequency variants than those present in release 22 of Hapmap project (296 SNPs vs. 40 SNPs), including a much larger fraction of variants with minor allele frequency (MAF) < 0.01 (154 vs. 0), with 0.01 < MAF < 0.05 (28 vs. 3), and with MAF > 0.05 (114 vs. 37). We chose 14 haplotype-tagging SNPs (htSNPs) from these variants (Supplementary Figure 1), and genotyped them in the Sample Set 1, including 1,299 cases and 1,067 controls of Southern Chinese ancestry.

**Figure 1:**
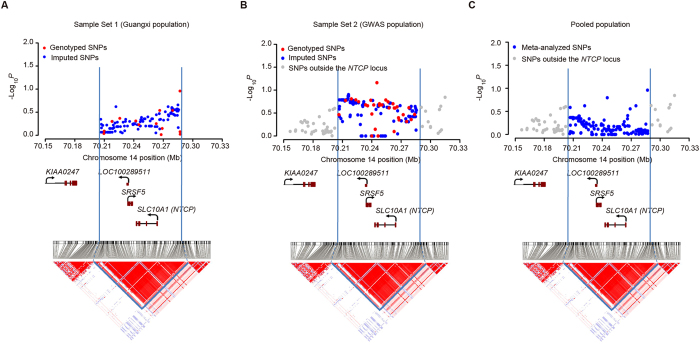
The association results of genotyped and imputed SNPs in the Sample Set 1 (Guangxi population), the Sample Set 2 (GWAS population), and the pooled population. SNPs surrounding the *SLC10A1* gene are plotted with their *P* values (shown as −log_10_ values) for additive model test as a function of genomic position (NCBI Build 37) in the the Sample Set 1 (**A**), the Sample Set 2 (**B**), and the pooled population by meta-analyses (**C**). Genomic locations of genes on the NCBI Build 37 human assembly were adapted from the University of California at Santa Cruz Genome Browser (http://genome.ucsc.edu/). The LD structure surrounding the *SLC10A1* gene in Chinese CHB and CHS samples of the 1000 Genomes Project was shown. Shading represents the magnitude and significance of pairwise LD (measured by *D*′), with a red-to-white gradient reflecting higher to lower LD values. The most intense red spots have a *D*′ = 1.

Then, genotype imputation was performed to generate additional genotypes in the locus across the *SLC10A1* gene, using the genotype data from CHB and CHS samples of the 1000 Genomes Project as reference. The use of this reference population for imputation permitted a comprehensive evaluation of common variants and some rarer variants at *SLC10A1* for susceptibility to persistent HBV infection. Genotype imputation was successful for 113 variants with most variants had MAF > 0.05, bringing the total number of variants evaluated in this haplotype block to 127 (Supplementary Table 2). By performing unconditional logistic regression analyses with adjustment for age and sex, we found little evidence of association between SNPs in the *SLC10A1* haplotype block and risk of persistent HBV infection in the Sample Set 1 (the lowest nominal *P* is 0.031; see Fig. 1 for additive model test, and Supplementary Figure 2 for dominant and recessive model tests). Therefore, common SNPs at the *SLC10A1* locus are probably not associated with risk of persistent HBV infection in the Sample Set 1.

We further validated whether or not common SNPs in this region were associated with risk of persistent HBV infection in the Sample Set 2 (GWAS population). As different genotyping arrays cover different SNPs at the *SLC10A1* locus in the five GWAS sub-populations of the Sample Set 2, we imputed the missing SNPs in every sub-population with reference haplotypes derived from all Chinese samples of the 1000 Genomes Project. Genotyping or imputation was successful for 132 variants in at least one sub-population, of which, 118 variants (89.4%) have been evaluated in the Sample Set 1 (Supplementary Table 2). By performing unconditional logistic regression analyses with adjustment for age and sex, we found none of the 132 variants were associated with risk of persistent HBV infection in GWAS-derived populations with the lowest nominal *P* value 0.069 (Fig. 1 and Supplementary Figure 2), consistent with the negative association results observed in the Sample Set 1.

To estimate the size of an overall effect, we performed meta-analyses of the two sample sets together by the Mantel-Haenszel test. The combined odds ratios (ORs) showed no evidence that any SNP was significantly associated with risk of persistent HBV infection with the lowest nominal *P* value 0.031 (Fig. 1 and Supplementary Figure 2). Therefore, common SNPs at the *SLC10A1* locus are unlikely to be associated with risk of persistent HBV infection in individuals of Southern Chinese ancestry.

To investigate whether or not multimarker haplotypes rather than single SNPs could mediate the association between *SLC10A1* and risk of persistent HBV infection, we moved on to perform haplotype analyses and found that 14 haplotypes were contained in this region. However, no haplotypes showed significant association in the Sample Set 1 after correction for multiple testing (the lowest nominal *P* value = 0.035, Supplementary Table 3).

Besides, when the analyses were stratified by age and sex, no significant associations were found between single SNPs or haplotypes in this region and the risk of persistent HBV infection in the Sample Set 1 (Supplementary Tables 4 and 5).

Taking together, these results indicated that neither common SNPs nor haplotypes at the *SLC10A1* locus were associated with risk of persistent HBV infection in subjects of Southern Chinese ancestry.

### Low-frequency non-silent variants at *SLC10A1*

We resequenced all exons and splicing regions of *SLC10A1* in 244 unrelated HBV carriers randomly selected from the Sample Set 1 (Supplementary Table 1B), to examine whether or not low-frequency non-silent variants at *SLC10A1* contribute to risk of persistent HBV infection. We only found one recurrent low-frequency non-silent variation (rs148467625) in this region, a nonsynonymous Isoleucine to Threonine change at residue 88 in exon 1. Reanalyzing the genotype data of the 1000 Genomes Project showed that the frequency of rs148467625 is low in the Southern Han Chinese population (MAF = 0.015), and this SNP may not exist in the Northern Han Chinese population and other ethnic populations. We then genotyped rs148467625 in the Sample Set 1 (Methods). The results showed that the MAF of rs148467625 in the Guangxi population is 0.015, similar to it’s MAF in the Southern Han Chinese population of the 1000 Genomes Project and the previous reports^21^,^24^. We found that this SNP showed no association with risk of persistent HBV infection in the Sample Set 1 (Supplementary Table 6). Together, these results suggest that low-frequency non-silent variants at *SLC10A1* may not individually represent a major risk factor for the risk of persistent HBV infection among Southern Chinese.

### Copy number variation (CNV) encompassing *SLC10A1*

CNV represents another important source of genetic variation complementary to SNP. Recently, CNVs have been shown to be important pathogenic factors in infectious diseases, including CNV of *CCL3L1* in human immunodeficiency virus type 1 (HIV-1) infection and CNV of the Fcγ-receptor genes in helminth infection, among many others^33^,^34^. To examine the role of CNV encompassing *SLC10A1* in the risk of persistent HBV infection, we screened the entire gene in the public database of genomic variants (DGV, http://projects.tcag.ca/variation/), but found no CNVs documented. The nearest CNVs flanking *SLC10A1* documented in the DGV database were two deletions with one located 7.4-Kb downstream and the other 14.3-Kb upstream of *SLC10A1* (Supplementary Figure 3). We also called CNVs in the Sample Set 2, which contains genome-wide genotyping data, but find no CNVs in *SLC10A1* or surrounding regions of 500-Kb in this relatively large dataset. These results suggest that CNV at the *SLC10A1* locus does not play an important role in predisposition to persistent HBV infection among Southern Chinese.

### Neutral theory test

Selection pressures due to infectious diseases have had a major impact on patterns of genetic variation within and between modern human populations^35^. Characterizing patterns of nucleotide diversity at candidate loci (e.g., *SLC10A1*) with susceptibility to persistent HBV infection, and testing for signatures of natural selection may be informative for identifying functional loci that are related to susceptibility to this infectious disease.

To quantify the intensity of natural selection for *SLC10A1*, we used the genotype data from CHB and CHS samples of the 1000 Genomes Project to perform five tests, including Tajima’s *D*, Fay and Wu’s *H*, Fu and Li’s *D*, HKA test, and F_*ST*_.

Both Tajima’s *D* and Fay and Wu’s *H* tests use summaries of the site frequency spectrum to identify signatures of selection. Fu and Li’s *D* statistic was used to compare the observed number of singleton polymorphisms with those expected under a neutral model. Although Fu and Li’s *D* test indicated a significant excess of singletons in this region in the Chinese Han population (*P* < 0.05), Tajima’s *D* and Fay and Wu’s *H* statistic suggested no statistically significant deviation from selective neutrality (Supplementary Table 7A). In addition, by the comparison of levels of polymorphism and divergence between *SLC10A1* and a locus evolving neutrally, the *DMD* intron 44, HKA test suggested no significant difference (*P* = 0.23). Furthermore, F_*ST*_ was calculated to test for differentiation between populations. The results showed that none of the SNPs at *SLC10A1* presented F_*ST*_ values greater than 0.5 (Supplementary Table 7B), indicating no evidence of significant differentiation between Chinese and other ethnic populations^36^.

Together, the results of neutral theory test suggest that the *SLC10A1* locus is probably under neutral selection, consistent with the results that none of the variants at the *SLC10A1* locus were associated with risk of persistent HBV infection.

### Expression quantitative trait locus (eQTL) analysis

Studies of the genetics of gene expression can identify expression SNPs (eSNPs) that explain variation in transcript abundance^37^. To investigate whether long-range regulation of eSNPs could mediate the association between *SLC10A1* and risk of persistent HBV infection, we performed cis- and trans-eQTL mapping, associating the *SLC10A1* expression trait with genotypes throughout the genome in the liver tissues of 31 PI samples (Supplementary Table 1C), by ANOVA model^38^.

At a threshold level of 0.001 for cis-eQTL within the 1-Mb upstream and 200-Kb downstream of *SLC10A1*, and 5.0 × 10^−8^ for trans-eQTL genome-wide^39^, no SNPs showed significant association with *SLC10A1* expression in cis-eQTL or trans-eQTL mapping (Supplementary Figure 4). Among them, ten cis-eSNPs showed marginally significance, with *P*_eQTL_ values between 0.001 and 0.01. However, in an effort to replicate the cis-eQTL results in data of the Genotype-Tissue Expression Project (GTEX) and other two eQTL studies of liver tissue (97, 427 and 532 samples, respectively)^40^,^41^, we found no cis-eSNPs at the *SLC10A1* locus significantly associated with the expression of *SLC10A1* (all *P* values > 0.05). Meanwhile, we analyzed the top signal of trans-eSNPs (rs3133759 and rs13255741 in 8q22), with *P*_eQTL_ values 5.8 × 10^−6^, in the Sample Set 2, but found no evidence of association of them with disease risk (*P* values for both SNPs > 0.05; Supplementary Table 8).

Taking together, these results suggest that potentially functional variants outside the *SLC10A1* locus conferring long-range regulation on *SLC10A1* expression may not play an important role in genetic susceptibility to persistent HBV infection among Southern Chinese.

## Discussion

As one-third of the individuals worldwide chronically infected with HBV reside in China, the genetic association study of the functional receptor for HBV, *SLC10A1*, bears important public health implications for control persistent HBV infection among Chinese. However, despite its excellent candidate gene status and previous reports, we found no strong evidence to show that common or rare genetic variants at the *SLC10A1* locus are associated with the risk of persistent HBV infection. There was, moreover, no evidence of a disease-associated haplotype in this region. CNV encompassing *SLC10A1* also does not play a major role in susceptibility to persistent HBV infection. Furthermore, the results of neutral theory test suggest that the *SLC10A1* locus is under neutral selection, consistent with the results that none of the variants at the *SLC10A1* locus were associated with the risk of persistent HBV infection. Finally, eQTL analyses indicated that genetic variation is not likely to be a major risk factor of this disease through long-range regulating *SLC10A1* expression. Overall, there is no evidence that genetic variation at *SLC10A1* is likely to be a major risk factor of persistent HBV infection among Southern Chinese. To our best knowledge, this is the first thorough research of the genetic association between *SLC10A1* and susceptibility to persistent HBV infection.

Our study had a number of strengths. First of all, in the present study, we selected the spontaneously recovered subjects (i.e., previously exposed patients) but not the unexposed subjects as controls, so our results may be more reliable than other reports in which general blood donors were recruited as controls. Because unexposed subjects remain at risk of acquiring persistent HBV infection, the inclusion of such controls limits the ability to compare frequency of variants in them to that in patients with established persistent HBV infection. Second, most of our HBV persistent carriers and subjects with nature HBV clearance came from systematic screening of HBV markers in large, population-based studies or routine physical examination in local hospitals and was matched on age and sex, which may have reduced potential selection bias. Finally, a relatively large sample size (totally 2,550 cases and 2,124 controls) in this study provided enough statistical power, >90% at significance level of 0.01, to detect an allele with a MAF of 0.20 that confers an additive 1.2-fold effect on disease risk (Supplementary Figure 5).

Previous studies have identified a number of functionally important variants at *SLC10A1*. Among them, a nonsynonymous SNP Ser267Phe, with MAF ~7.5% in people of Chinese origin and nearly absent in non-Asian ethnic populations, leads to a near complete loss of bile acid transport activity via loss of substrate specificity for bile acids^18^, and to reduced HBV infection^17^. Recently, two large cohort studies have reported that the Ser267Phe variant was associated with resistance to HBV infection among Chinese of Guangdong (under multiple models of inheritance) and Taiwan (only under the recessive model), respectively. However, in the present study with large number of participants recruited also in southern China, Ser267Phe was not significantly associated with risk of persistent HBV infection (the lowest *P* value = 0.031, before adjustment for multiple testing under the recessive model in the pooled population, Supplementary Table 2). Even if Ser267Phe is regarded as a protective factor against persistent HBV infection when considering the marginal association (*P* = 0.031), the population attributable fraction (PAF) indicated that only 5.5% (95% CI, 0.15–39.2%) of the decrease in the risk of persistent HBV infection can be attributed to the protective effect of the Ser/Ser genotype in this study (methods). Therefore, the inconsistent results across studies suggest that Ser267Phe may be not involved, or at best play a minor role, in decreased susceptibility to persistent HBV infection.

Another naturally occurring nonsynonymous variation is Ile279Thr with allele frequencies of 0.5% in people of Chinese origin and almost absent in other ethnic populations, demonstrating a significant reduction in taurocholate uptake although not to the degree seen with Ser267Phe^18^. However, Ile279Thr was seen only once from a total of 370 DNA samples tested in the original research, and absent in the 669 samples in Pan’s study (including 366 Chinese)^19^, 1,092 samples in the 1000 Genomes Project (including 197 Chinese) and our 244 Chinese cases resequenced for variants in *SLC10A1* exons.

A recent study has indicated that residues between 157 and 165 of NTCP are crucial for binding to the receptor-binding region of the pre-S1 domain of the L protein of HBV, and critically contribute to NTCP-mediated HBV and HDV infections^14^. Therefore, it is reasonable that functional genetic variants within these residues would influence the interaction between HBV and its receptor NTCP, which may in turn result in genotype-dependent differences in susceptibility to persistent HBV infection. However, we screened the 1000 Genomes Project database and our own sequencing database, only to find no variants in this region in Chinese population of the *SLC10A1* gene.

Overall, focusing on previously reported functional variants at *SLC10A1* also showed no evidence of a major role of *SLC10A1* variants in susceptibility to persistent HBV infection.

There are several possible reasons for our negative association results. First, inadequate power may be an explanation of our results. Although we have relatively high power to detect common alleles with moderate or greater effects as mentioned above, we failed to find any rare functional variants in *SLC10A1* with significant association, which, to some extent, showed that rare variants in *SLC10A1* may not contribute to the risk of persistent HBV infection. However, more samples need to be sequenced for *SLC10A1* rare variants to obtain more credible conclusion.

Alternatively, differences in the genetic effect among ethnic groups may be another explanation. There may be a small, population-specific effect of *SLC10A1* polymorphisms on the risk of persistent HBV infection. This might occur if there were population differences in linkage disequilibrium (LD) pattern or allele frequencies of *SLC10A1*. Indeed, the allele and genotype frequencies of the *SLC10A1* polymorphisms vary with ethnicity. For instance, in the 1068 control subjects of the Sample Set 1 (Southern Chinese), we found that the frequencies of the 267Ser allele and Ser/Ser genotype were 0.10 and 0.0077, while in non-Asian population of the 1000 Genomes Project, the frequency of the 267Ser allele was extremely low, being 0 and 0.0016 in European and African populations, respectively. Furthermore, no 267 Ser alleles were found in more than 6000 non-Asian samples of the NHLBI Exome Sequencing Project (Data release: ESP6500SI-V2, http://evs.gs.washington.edu/EVS/). Thus, ethnic variation in the *SLC10A1* genotype distribution warrants additional comparative studies in other populations of different ancestry, such as Caucasians and Africans, to confirm our results.

Finally, it is not surprising that variants at *SLC10A1*, a functional receptor for HBV, may not be associated with risk of persistent HBV infection. Previous studies have shown that polymorphisms in virus receptors may not be always associated with virus clearance, such as CD81 and LDLR, receptors for HCV^42^, and CLEC4M, receptor for SARS-CoV^43^,^44^. It remains to be determined if variants of other molecule (s) as a coreceptor (s) or receptor component (s) additional to NTCP contributing to the cellular entry of HBV, and if variants of other host factors like those that have been shown to involve in infections of other viruses, would contribute to risk of persistent HBV infection.

In conclusion, we for the first time performed a thorough research of the genetic association between *SLC10A1* variants and susceptibility to persistent HBV infection. Our results suggest that common and rare alleles or CNVs across the *SLC10A1* locus do not confer a major risk of persistent HBV infection among Southern Chinese. However, additional studies from larger cohorts among Northern Chinese, and from diverse ethnic groups, are warranted before the importance of *SLC10A1* variants in risk of persistent HBV infection can be fully ascertained.

## Materials and Methods

This study was approved by the Medical Ethical Committee of Beijing Institute of Radiation Medicine (Beijing, China). The written informed consents were obtained from all the participants enrolled in the study. And personal information on demographic factors was collected by structured questionnaire. All experiment methods were performed in accordance with the relevant guidelines and regulations.

### Study samples

This study included two independent case-control populations, namely the Sample Set 1 (Guangxi population) and the Sample Set 2 (GWAS population), totally consisting of 2,550 cases and 2,124 controls (Supplementary Table 1). All the participants were ethnic adult Chinese, and recruited in southern China, including Guangxi, Jiangsu, and Guangdong provinces. The inclusion and exclusion criteria for the cases and controls were described in detail previously^45^. Briefly, subjects who had been positive for both hepatitis B surface antigen (HBsAg) and antibody to hepatitis B core antigen (anti-HBc) immunoglobulin G (IgG) for at least 6 months were defined as cases (the persistently HBV infected subjects, PIs). Those who were negative for HBsAg and positive for antibody to hepatitis B surface antigen (anti-HBs) and anti-HBc IgG, were defined as controls (spontaneously recovered subjects, SRs), with frequency matching to the cases on sex and age (±5 years). Individuals were excluded if they reported being vaccinated with HBV vaccine. Furthermore, all the individuals who were positive for anti-HBs and negative for anti-HBc IgG (may have vaccination history) were excluded. All the recruited subjects were self-reported Han Chinese and had no serological evidence for hepatitis C virus, hepatitis D virus, and HIV co-infection.

### The Sample Set 1 (Guangxi population)

This population was recruited as part of a community cancer screening program (Yulin City, Guangxi province, China) for early detection of cancer performed in between February 2011 and October 2012. Of them, 1,299 and 1,067 were determined as PIs and SRs, respectively^10^. The male/female ratio and the mean age (s.d.) are 1.6 (806/493) and 38.2 (10.7) years old for PIs, and 1.6 (651/416) and 38.4 (12.4) years old for SRs, respectively (Supplementary Table 1A).

### The Sample Set 2 (GWAS population)

The genotype data of the Sample Set 2 were derived from previously published and in-house GWAS data^29^,^30^,^31^,^32^, as described in detail in our previous study^10^. Briefly, the data of these GWASs totally consist of 12,027 individuals of Chinese ancestry, who have been genotyped by various SNP arrays^10^. By screening for HBV markers in the plasma of subjects whose plasma samples were available, we finally determined that the Sample Set 2 contains five independent GWAS sub-populations providing totally 1,251 cases and 1,057 controls (Supplementary Table 1A). Patients with cancer other than HBV-related HCC were excluded from screening.

GWAS sub-population 1 contains 286 PIs and 656 SRs identified from 1,999 cancer-free subjects in the GWAS on serum levels of complement C3 and C4 (genotyped by Illumina Omini one array)^29^. All the subjects are males and the mean ages (s.d.) are 37.2 (10.1) and 37.1 (10.8) years old, for PIs and SRs, respectively. GWAS sub-population 2 contains all of the 707 PIs (348 PIs with HBV-related HCC and 359 cancer-free PIs) derived from the GWAS on HBV-related HCC genotyped by Affymatrix SNP 5.0 array^30^. The male/female ratio and the mean age (s.d.) of these cases are 6.5 (613/94) and 43.7 (11.6) years old, respectively. GWAS sub-population 3 contains 78 PIs and 74 SRs identified from 436 cancer-free subjects genotyped by Illumina Human Omni Zhonghua BeadChip (in-house data)^10^. The male/female ratio and the mean age (s.d.) are 0.3 (20/58) and 62.8 (8.3) years old for PIs, and 0.8 (33/41) and 66.0 (7.2) years old for SRs, respectively. All of the above three GWAS sub-populations were recruited from Guangxi province, thus were combined and named as “GWAS-Guangxi population” (Supplementary Table 1A).

GWAS population 4 contains 91 PIs and 203 SRs identified from 3,077 cancer-free subjects in the GWAS on lung cancer (genotyped by Affymatrix SNP 6.0 array)^31^. The male/female ratio and the mean age (s.d.) are 4.4 (74/17) and 56.6 (10.0) years old for PIs, and 2.3 (142/61) and 57.5 (9.7) years old for SRs, respectively. This sub-population was recruited from Jiangsu province, thus was named as “GWAS-Jiangsu population” (Supplementary Table 1A).

GWAS population 5 contains 89 PIs and 124 SRs identified from 1,894 cancer-free subjects in the GWAS on nasopharyngeal carcinoma (NPC) genotyped by Illumina Human610-Quad BeadChip^32^. The male/female ratio and the mean age (s.d.) are 2.9 (66/23) and 46.3 (11.1) years old for PIs, 2.8 (91/33) and 47.7 (13.0) years old for SRs, respectively. This sub-population was recruited from Guangdong province, thus was named as “GWAS-Guangdong population” (Supplementary Table 1A).

### htSNPs selection, genotyping and quality control

htSNPs selection were performed based on genotyped SNPs in the CHB and CHS samples of the 1000 Genomes Project. We defined LD blocks as the region between the boundaries of two consecutive recombination hotspots and 14 htSNPs across the *SLC10A1* gene were selected with Haploview^46^ with the default ‘confidence interval’ option (Supplementary Figure 1). Then the 14 htSNPs together with rs148467625, were genotyped in the Sample Set 1 (Guangxi population) using MassArray platform of Sequenom with primers listed in Supplementary Table 9. All of the 15 SNPs in the Sample Set 1 satisfied the quality control criteria, with a mean call rate of 98.3% in all samples and with no evidence of departure from Hardy-Weinberg equilibrium (HWE) in the controls (*P*_HWE_ ≥ 0.01). Quality control was conducted using the program PLINK. A 5% random sample was tested by Sanger sequencing, and the reproducibility was 99.9%.

### SNP imputation

To generate additional genotypes in the region across the *SLC10A1* gene and increase the number of overlapping SNPs between data sets, we performed imputation on the GWAS data sets and Guangxi population using SHAPEIT^47^ and IMPUTE2^48^, using the genotype data from CHB and CHS samples of the 1000 Genomes Project as reference. SNPs with low imputation quality (info score <0.6 for IMPUTE2) were removed. For all data sets, cases and controls were imputed together.

### CNV detection

CNV detection was performed in the Sample Set 2 (GWAS population), for whom microarray-based genome-wide SNP genotyping was done using five different assays. Partek and Birdeye^49^ were used for CNV detection with genotype data of Affymatrix Genome-Wide Human SNP Array 5.0 (GWAS sub-population 2) and 6.0 (GWAS sub-population 4). Samples with mean signal intensity deviating from mean signal intensity of all samples by three standard variation, or with median signal intensity deviating from median signal intensity of all samples by 1.5 interquartile range (IQR) were excluded.

PennCNV^50^ and QuantiSNP^51^ were used for CNV detection with genotype data of Illumina microarray chips (Omni one for GWAS sub-population 1, Omni Zhonghua for GWAS sub-population 3, and 610-Quad for GWAS sub-population 5). Samples with LogR standard deviation >0.35, BAF drift >0.01 or Waviness factor (WF) deviating from 0 by >0.04 after correction were excluded. Furthermore, samples with an excessive number of CNVs (≥1.5 IQR above the median number of CNVs per individual) were excluded.

Overall, 1,076 cases and 838 controls of the GWAS population were used in the final CNV analysis. CNVs smaller than 300-bp or covered by less than three probes were excluded.

### Exon resequencing and variants identification

We used conventional PCR and Sanger sequencing to screen all protein-coding exons and splicing regions of *SLC10A1* for 40 unrelated PI samples (Supplementary Table 1B). Each fragment amplified by PCR from genomic DNA was sequenced on both strands with an ABI Prism 3700 sequencer. The Phred-Phrap-Consed-PolyPhred package (http://www.genome.washington.edu/) was used to assemble the sequences and identify variants.

### Neutral theory test

To test for differentiation between populations, three measures of diversity were computed for Chinese samples: (1) Watterson’s θ_*w*_ based on the number of segregating sites in the sample, an estimate of the expected per-site nucleotide heterozygosity, theoretically equal to the neutral mutation parameter 4*N*_*e*_*μ*; (2) π, the direct estimate of per-site heterozygosity derived from the average number of pairwise sequence differences in the sample; and (3) θ_*H*_, a summary that gives more weight to high frequency-derived variants. To test whether the frequency spectrum of mutations conformed to the expectations of the standard neutral model, we calculated the values of two test statistics: (1) Tajima’s *D* statistic, which considers the difference between θ_*w*_ and π, and (2) Fay and Wu’s *H* statistic, which considers the difference between θ_*H*_ and π. Under neutrality, the two test statistics should be close to 0. Furthermore, Fu and Li’s *D* statistic was used to compare the observed number of singleton polymorphisms with those expected under a neutral model.

Significance values for each of the above three test statistics were estimated from 10^4^ coalescent simulations of a Wright-Fisher equilibrium model that condition on the sample size and level of polymorphism as the observed data, with no recombination, using DnaSP Software^52^ and Fay’s *H*-test (http://www.genetics.wustl.edu/jflab/htest.html), respectively.

The Hudson/Kreitman/Aguade (HKA) test was used to compare diversity patterns in the *SLC10A1* region with diversity patterns found at the *DMD* intron 44 locus which is assumed to evolve neutrally^53^, using DnaSP Software^52^.

F_*ST*_, which reflects differences in allele frequencies among samples and increases as allele frequency differences between population samples become more pronounced, was calculated using R software (http://www.r-project.org/).

### eQTL analysis

Cis- and trans- eQTL mapping were performed by ANOVA model^38^ to associate the *SLC10A1* expression trait with genotypes throughout the genome in the liver tissues of 31 PI samples used in our previous study^10^. The genotypes of these subjects were generated using the Affymetrix Genome-Wide Human SNP Array 6.0, and the mRNA expression profiles of tissues from them were obtained using the Affymetrix Human Exon 1.0 ST arrays (microarray GEO number: GSE74925). The mRNA expression levels were log2 transformed. The intensity of all gene array experiments were normalized together using the Robust Multiarray Averaging (RMA) method. A genome-wide significance threshold level of 5.0 × 10^−8^ is applied for trans-eQTL^39^ and a threshold level of 0.001 is set for cis-eQTL within the 1-Mb upstream and 200-Kb downstream of *SLC10A1*.

### Calculation of population attributable fraction (PAF)

PAF can be calculated with the formula of f (RR-1)/[1 + f (RR-1)], where f is the population exposure rate, and RR is the relative risk^54^. For Ser267Phe, the f is the frequency of the Ser/Ser genotype (6.5%), and the protective effect (RR) of Ser/Ser is 10.0 (95% CI, 1.23–100.0) under the recessive model in the pooled population (Supplementary Table 2).

### Statistical analyses

The power to detect genetic effect sizes was calculated by Power for Genetic Association Analyses (PGA), which is a package of algorithms and graphical user interfaces developed in Matlab for power and sample size calculation under various genetic models and statistical constraints^55^. The association between SNP genotype and disease status was evaluated under three models (additive, dominant and recessive) by unconditional logistic regression analyses with adjustment for the confounding factors sex and age. PLINK software was used to obtain association *P*-values and to calculate odds ratios (ORs) and 95% confidence interval (CI). Meta-analyses of data generated from the Sample Set 1 and Sample Set 2 were conducted to assess pooled genetic effects. Meta-analyses were performed using the fixed-effects model (Mantel-Haenszel model) as heterogeneity was low (the value of the I-squared statistic was below 50%). The regional plot was plotted using an in-house program. To analyze the association of haplotypes, we used the haplo.stats package (version 1.7.7)^56^ of R. *P* values for each haplotype were given by the results of a score test, and ORs and 95% CIs were calculated from coefficients of GLM model. ORs of each haplotype were calculated relative to the major haplotype. All of these statistical values were calculated by the function haplo.cc of the haplo.stats package.

## Additional Information

**How to cite this article**: Zhang, Y. *et al*. Comprehensive assessment showed no association of variants at the *SLC10A1* locus with susceptibility to persistent HBV infection among Southern Chinese. *Sci. Rep.* **7**, 46490; doi: 10.1038/srep46490 (2017).

**Publisher's note:** Springer Nature remains neutral with regard to jurisdictional claims in published maps and institutional affiliations.

## Supplementary Material

Supplementary Information

Supplementary Tables
