# Supplementary material for: Comprehensive assessment showed no associations of variants at the SLC10A1 locus with susceptibility to persistent HBV infection among Southern Chinese
Source: Sci Rep. 2017 Apr 21;7:46490. doi: 10.1038/srep46490 (PMC5399367; doi:10.1038/srep46490)
Supplement: Supplementary Information [file srep46490-s1.pdf]

# Supplementary Materials

## Comprehensive assessment showed no association of variants at the *SLC10A1* locus with susceptibility to persistent HBV infection among Southern Chinese

**Short title: *SLC10A1* variants and persistent HBV infection**

Ying Zhang[1,2,6,7,*], Yuanfeng Li[2,6,7,*], Miantao Wu[9,*], Pengbo Cao[2,6,7], Xiaomin Liu[10], Qian Ren[2,6,7], Yun Zhai[2,6,7], Bobo Xie[2,6,7], Yanling Hu[3], Zhibin Hu[4], Jinxin Bei[5], Jie Ping[2,6,7], Xinyi Liu[2,6,7], Yinghua Yu[8], Bingqian Guo[2,6,7], Hui Lu[2,6,7], Guanjun Liu[8], Haitao Zhang[2,6,7], Ying Cui[8], Zengnan Mo[3], Hongbing Shen[4],Yi-Xin Zeng[5], Fuchu He[1,2,6,7], Hongxing Zhang[2,6,7], and Gangqiao Zhou[2,6,7]

[1]School of Life Sciences, Tsinghua University, Beijing, China;

[2] State Key Laboratory of Proteomics, Beijing Proteome Research Center, Beijing Institute of Radiation Medicine, Beijing, China;

[3]Center for Genomic and Personalized Medicine, Guangxi Medical University, Nanning, Guangxi, China;

[4]Department of Epidemiology and Biostatistics, MOE Key Laboratory of Modern Toxicology, School of Public Health, Nanjing Medical University, Nanjing, China;

[5]State Key Laboratory of Oncology in Southern China, Guangzhou, China;

[6]National Engineering Research Center for Protein Drugs, Beijing, China;

[7]National Center for Protein Sciences Beijing, Beijing, China;

[8]Affiliated Cancer Hospital of Guangxi Medical University, Nanning, Guangxi, China;

[9] State Key Laboratory of Oncology in South China, Collaborative Innovation Center for Cancer Medicine, Sun Yat-sen University Cancer Center, Guangzhou, China;

[10] Department of Laboratory Medicine, Sun Yat-sen University Cancer Center, Guangzhou, China.

*These authors contributed equally to this work.

**Correspondence should be addressed to:**

Dr. Gangqiao Zhou, State Key Laboratory of Proteomics, Beijing Proteome Research Center, Beijing Institute of Radiation Medicine, 27 Taiping Road, Beijing 100850, P. R. China. E-mail: zhougq114@126.com; Phone: 86-10-66931204.

or

Dr. Hongxing Zhang, State Key Laboratory of Proteomics, Beijing Proteome Research Center, Beijing Institute of Radiation Medicine, 27 Taiping Road, Beijing 100850, P. R. China. E-mail: zhanghx08@126.com; Phone &fax: 86-10-61777099.

or

Dr. Fuchu He, State Key Laboratory of Proteomics, Beijing Proteome Research Center, Beijing Institute of Radiation Medicine, 27 Taiping Road, Beijing 100850, P. R. China. E-mail: hefc@nic.bmi.ac.cn; Phone &fax: 86-10-68177417.

## Index

**Supplementary Tables (see the attached Excel file):**

**Supplementary Table 1:** Selected characteristics of the subjects involved in the present study.

**Supplementary Table 2:** The association results of genotyped and imputed SNPs in the Sample Set 1 (Guangxi population), the Sample Set 2 (GWAS population), and the pooled population.

**Supplementary Table 3:** The association results of haplotypes in the Sample Set 1.

**Supplementary Table 4:** Stratification analysis for genotyped and imputed SNPs by sex and age at diagnosis in the Sample Set 1.

**Supplementary Table 5:** Stratification analysis for haplotypes by sex and age at diagnosis in the Sample Set 1.

**Supplementary Table 6:** The association results of the low-frequency non-silent variation rs148467625 in the Sample Set 1.

**Supplementary Table 7:** Neutral theory test.

**Supplementary Table 8:** Association results of rs3133759 and rs13255741 in the Sample Set 2.

**Supplementary Table 9:** Primers used for genotyping the SNPs in *SLC10A1* with the Sequenom MassArray platform.

**Supplementary Figures:**

**Supplementary Figure 1:** Fourteen haplotype-tagging SNPs (htSNPs) in the *SLC10A1* region.

**Supplementary Figure 2:** The association results of genotyped and imputed SNPs in the Sample Set 1 (Guangxi population), the Sample Set 2 (GWAS population), and the pooled population.

**Supplementary Figure 3:** The known CNVs covering *SLC10A1* and its flanking region.

**Supplementary Figure 4:** Graphical scheme of eQTL obtained by ANOVA analysis in the liver tissues of 31 persistent HBV infected subjects (PIs).

**Supplementary Figure 5:** Power to detect a genetic effect of various sizes (OR = 1.1, 1.2, 1.3, or 1.4) versus study sample size.

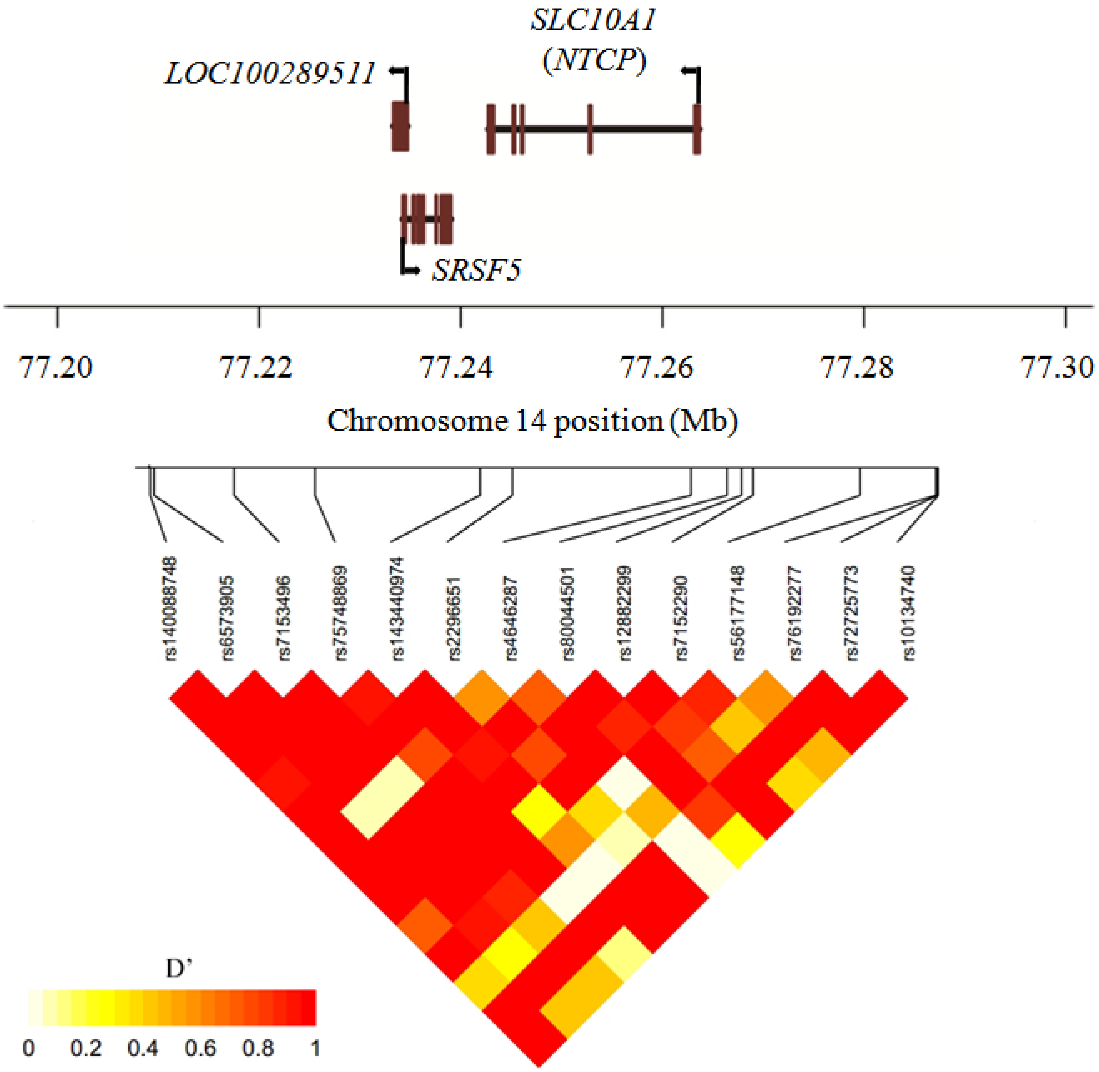


**Supplementary Figure 1:** Fourteen haplotype-tagging SNPs (htSNPs) in the *SLC10A1* region. Genomic locations of genes on the NCBI Build 37 human assembly were adapted from the University of California at Santa Cruz Genome Browser (http://genome.ucsc.edu/). The LD structure surrounding the *SLC10A1* gene in Chinese CHB and CHS samples of the 1000 Genomes Project was shown. Shading represents the magnitude and significance of pairwise LD (measured by *D'*), with a red-to-white gradient reflecting higher to lower LD values.

A.

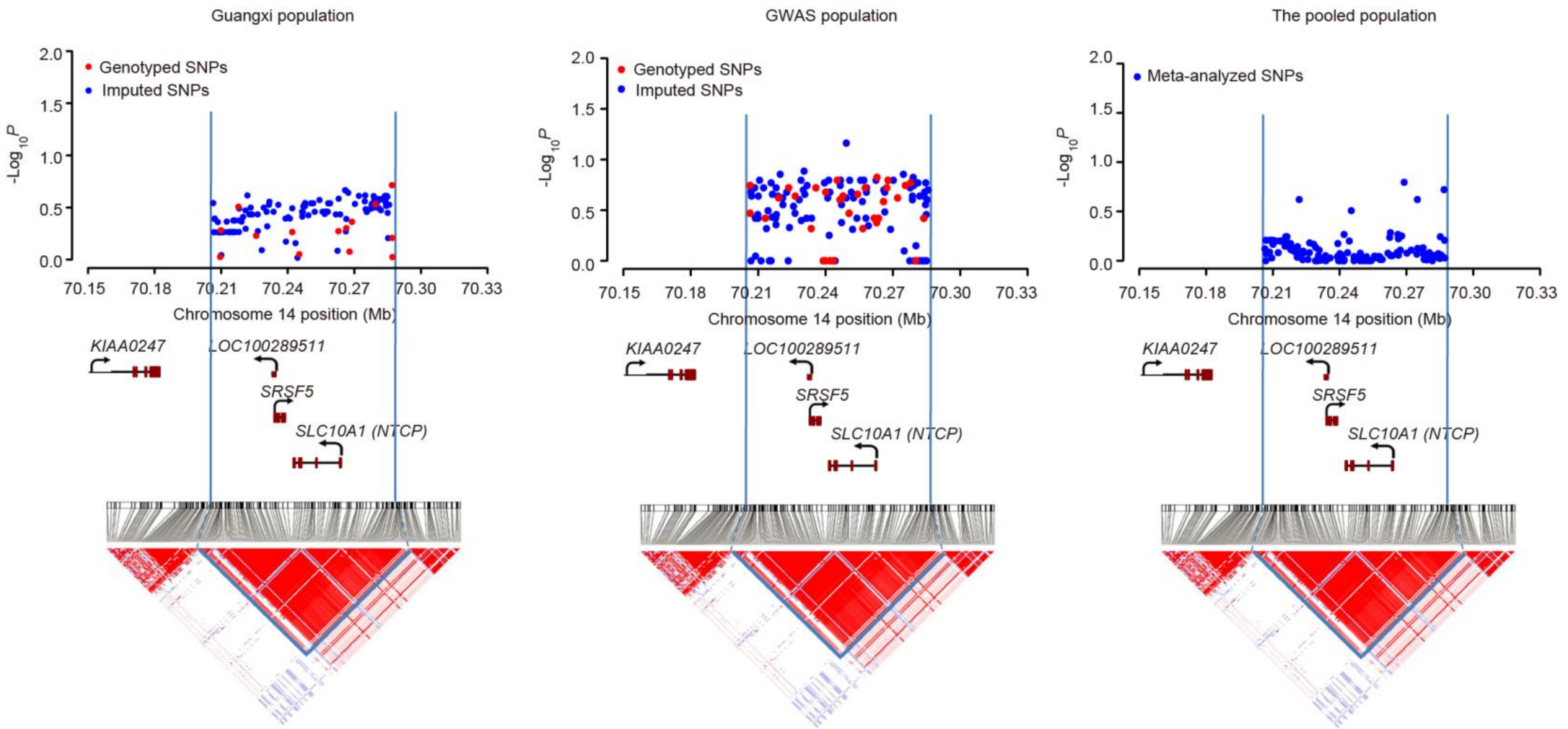
Guangxi population
GWAS population
The pooled population
Genotyped SNPs
Imputed SNPs
Meta-analyzed SNPs
-Log10P
2.0
1.5
1.0
0.5
0.0
70.15
70.18
70.21
70.24
70.27
70.30
70.33
Chromosome 14 position (Mb)
KIAA0247
LOC100289511
SRSF5
SLC10A1 (NTCP)

B.

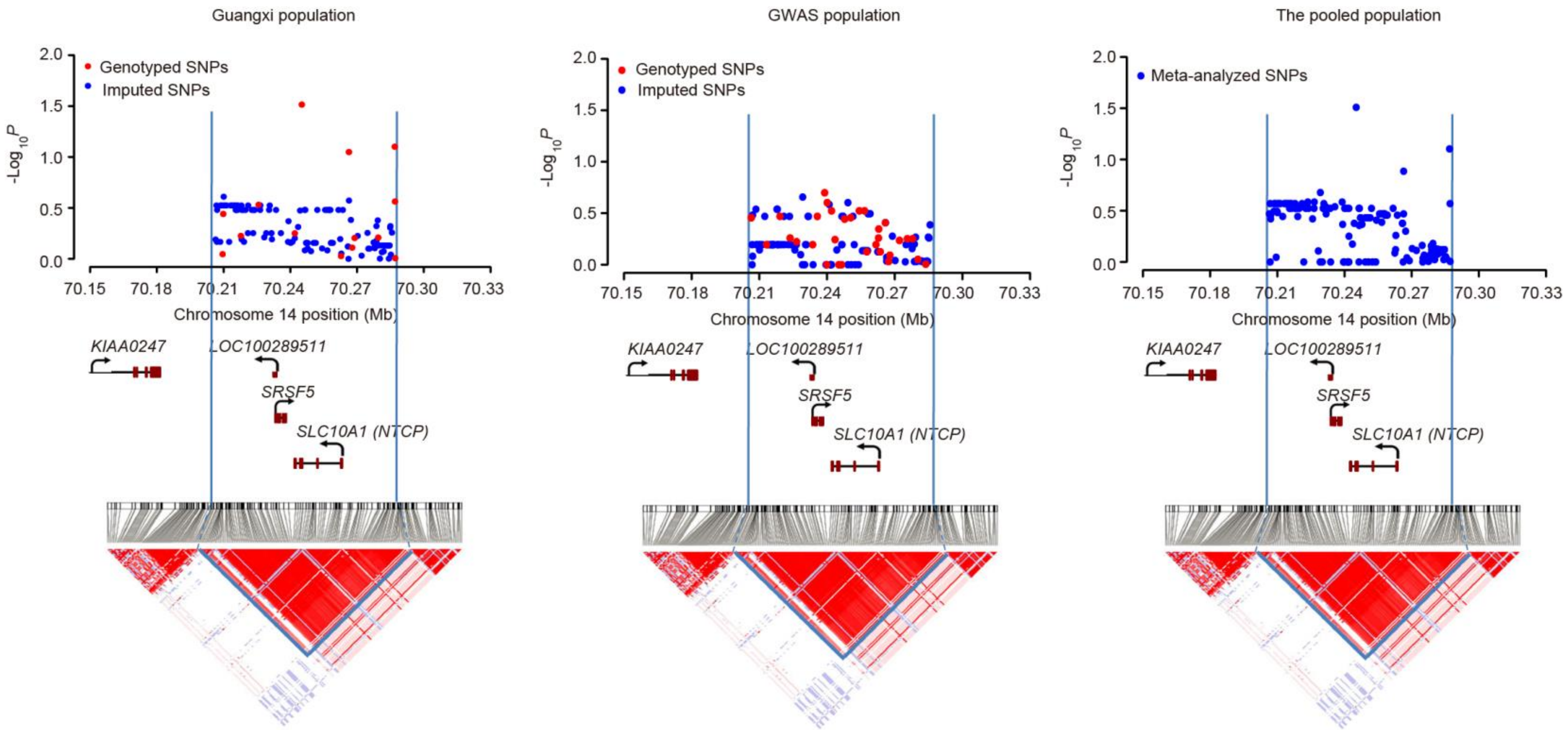
Guangxi population
GWAS population
The pooled population
Genotyped SNPs
Imputed SNPs
Meta-analyzed SNPs
-Log10P
2.0
1.5
1.0
0.5
0.0
70.15
70.18
70.21
70.24
70.27
70.30
70.33
Chromosome 14 position (Mb)
KIAA0247
LOC100289511
SRSF5
SLC10A1 (NTCP)

**Supplementary Figure 2:** The association results of genotyped and imputed SNPs in the Sample Set 1 (Guangxi population), the Sample Set 2 (GWAS population), and the pooled population. SNPs surrounding *SLC10A1* are plotted with their *P* values (shown as $-\log_{10}$ values) for dominant (A), and recessive (B) model tests as a function of genomic position (NCBI Build 37) in the Sample Set 1, the Sample Set 2, and the pooled population by meta-analyses. Genomic locations of genes on the NCBI Build 37 human assembly were adapted from the University of California at Santa Cruz Genome Browser (http://genome.ucsc.edu/). The LD structure surrounding the *SLC10A1* gene in Chinese CHB and CHS samples of the 1000 Genomes Project was shown. Shading represents the magnitude and significance of pairwise LD (measured by *D'*), with a red-to-white gradient reflecting higher to lower LD values. The most intense red spots have a *D'*= 1.

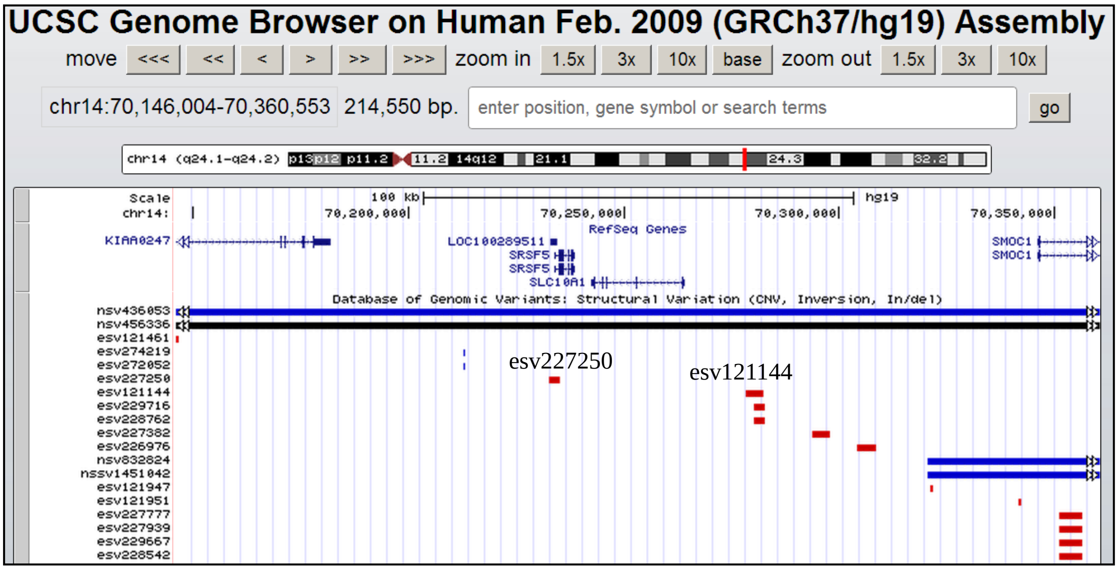


**Supplementary Figure 3:** The known CNVs covering *SLC10A1* and its flanking region. The nearest CNVs flanking *SLC10A1* documented in the database of genomic variants (DGV) were two deletions, of which one (esv227250, chr14:70232648-70235147, 2.5-Kb in length) located 7.4-Kb downstream and the other (esv121144, chr14:70278335-70282507, 4.2-Kb in length) 14.3-Kb upstream of *SLC10A1*. The figure was adapted from the University of California at Santa Cruz Genome Browser (http://genome.ucsc.edu/).

A.

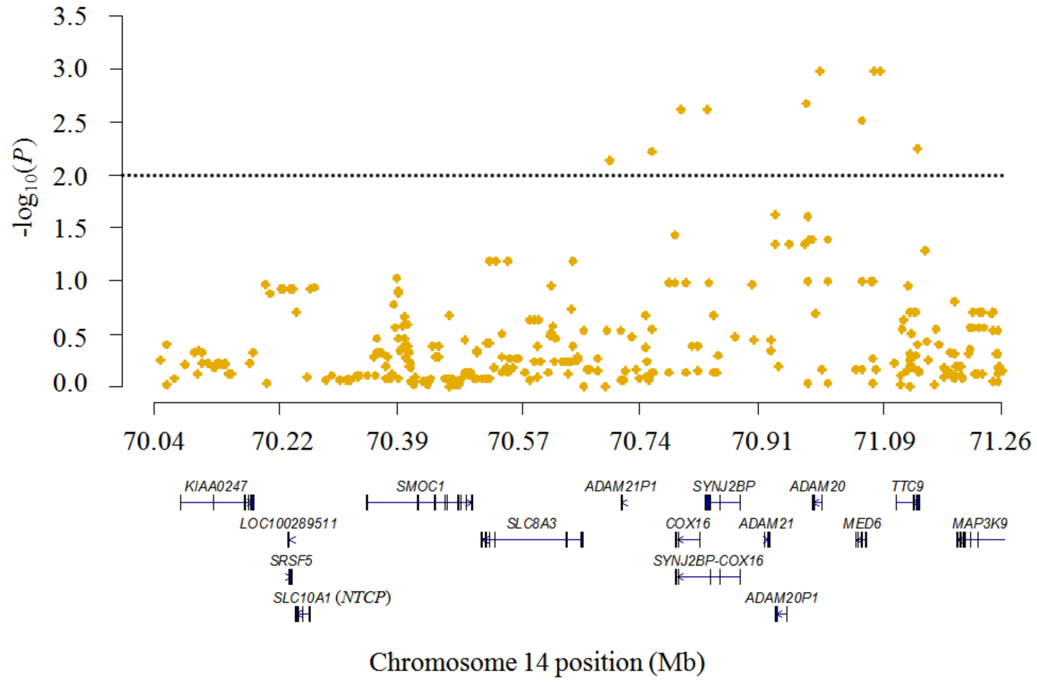


B.

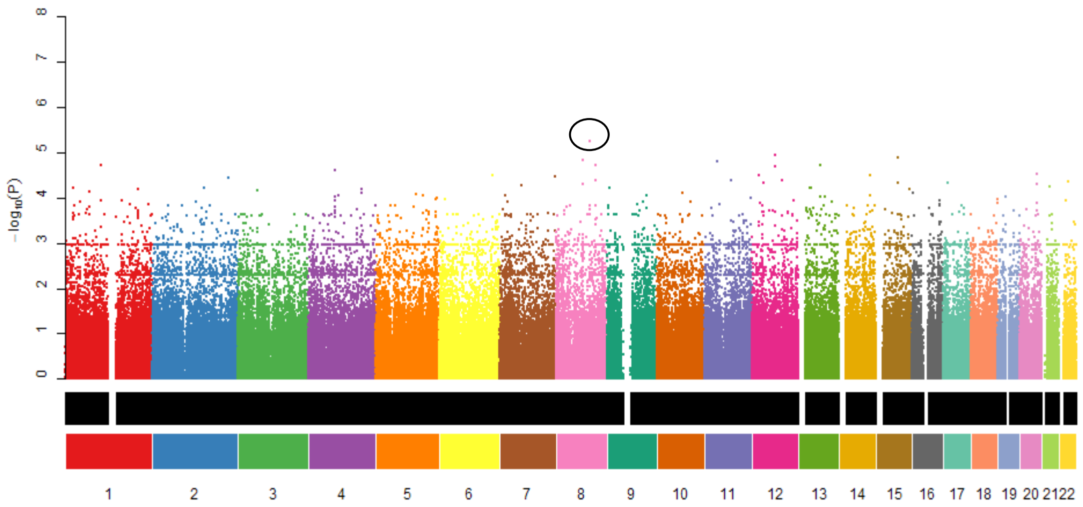


**Supplementary Figure 4:** Graphical scheme of eQTL obtained by ANOVA analysis in the liver tissues of 31 persistent HBV infected subjects (PIs). A, Cis-eQTL analysis indicated that no SNPs within the 1-Mb upstream and 200-kb downstream of *SLC10A1* showed significant association (*P*<0.001) with *SLC10A1* expression, with

10 SNPs showing marginally significance (*P*<0.01). B, Trans-eQTL analysis indicated that no genome-wide significant trans-eSNPs ($P<5.0\times10^{-8}$) were found, with only two SNPs rs3133759 and rs13255741 (the circled points) showing nominal significance ($P= 5.8\times10^{-6}$). The x-axis represents genomic position (NCBI Build 37), and the y-axis shows $-\log_{10}(P)$. Within each chromosome shown on the x-axis, the data are plotted from the p-ter end.

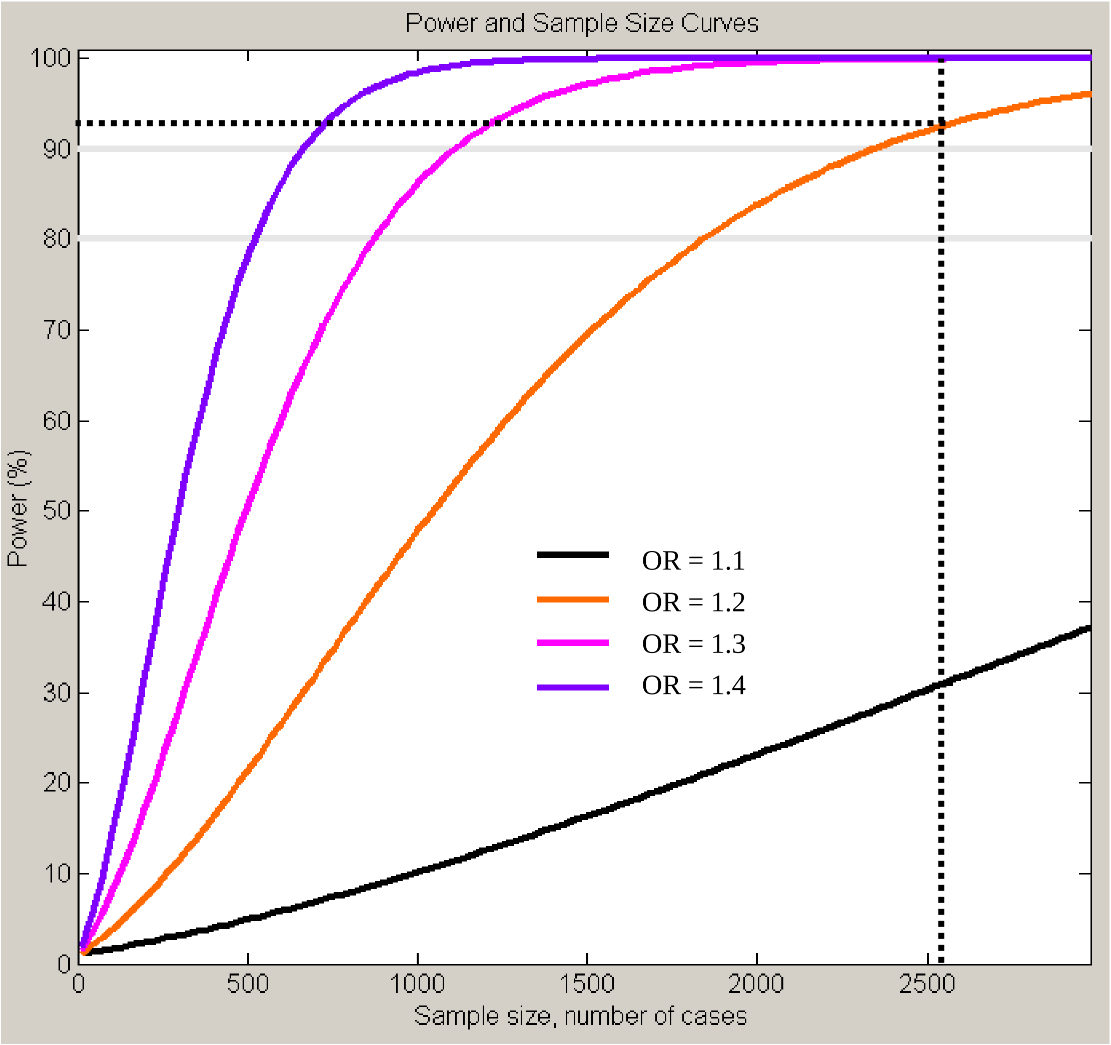


**Supplementary Figure 5:** Power to detect a genetic effect of various sizes (OR = 1.1, 1.2, 1.3, or 1.4) versus study sample size. Power is reported here as the probability of SNPs to be identified in a scan. Vertical and horizontal dashed lines show that the power of our pooled population (totally 2,550 cases and 2,124 controls), at significance level of 0.01, to detect an allele with a minor allele frequency (MAF) of 0.20 that confers an additive 1.2-fold effect on risk of persistent HBV infection, was estimated to be ~92%.
